# SARS-CoV-2 reinfections in a US university setting, Fall 2020 to Spring 2021

**DOI:** 10.1186/s12879-022-07578-x

**Published:** 2022-07-04

**Authors:** Molly Rosenberg, Chen Chen, Lilian Golzarri-Arroyo, Aaron Carroll, Nir Menachemi, Christina Ludema

**Affiliations:** 1 Department of Epidemiology and Biostatistics, Indiana University School of Public Health-Bloomington, 1025 E. 7th St., Bloomington, IN 47405 USA; 2 Biostatistics Consulting Center, Indiana University School of Public Health-Bloomington, Bloomington, IN USA; 3 Pediatric and Adolescent Comparative Effectiveness Research, Indiana University School of Medicine, Indianapolis, IN USA; 4 Department of Health Policy and Management, Indiana University Fairbanks School of Public Health at IUPUI, Indianapolis, IN USA

**Keywords:** SARS-CoV-2, COVID-19, Reinfection, Asymptomatic infection, University settings

## Abstract

**Background:**

SARS-CoV-2 reinfections are a public health concern because of the potential for transmission and clinical disease, and because of our limited understanding of whether and how well an infection confers protection against subsequent infections. Despite the public health importance, few studies have reported rigorous estimates of reinfection risk.

**Methods:**

Leveraging Indiana University’s comprehensive testing program to identify both asymptomatic and symptomatic SARS-CoV-2 cases, we estimated the incidence of SARS-CoV-2 reinfection among students, faculty, and staff across the 2020–2021 academic year. We contextualized the reinfection data with information on key covariates: age, sex, Greek organization membership, student vs faculty/staff affiliation, and testing type.

**Results:**

Among 12,272 people with primary infections, we found a low level of SARS-CoV-2 reinfections (0.6%; 0.4 per 10,000 person-days). We observed higher risk for SARS-CoV-2 reinfections in Greek-affiliated students.

**Conclusions:**

We found evidence for low levels of SARS-CoV-2 reinfection in a large multi-campus university population during a time-period prior to widespread COVID-19 vaccination.

**Supplementary Information:**

The online version contains supplementary material available at 10.1186/s12879-022-07578-x.

## Introduction

The first global case of SARS-CoV-2 reinfection was documented in August 2020 [1], with additional reports published soon after [2, 3]. Reinfections are a significant public health concern because they establish whether SARS-CoV-2 is able to evade immunity [4]. Characterizing reinfections is important to inform our understanding of whether and how well an infection confers protection against subsequent infections, which will improve our ability to accurately project future pandemic trajectories [5]. Filling reinfection knowledge gaps is especially important to improve guidance for undervaccinated individuals who may be making incorrect assumptions about their immune status from prior infections [6]. Of course, as with primary SARS-CoV-2 infections, reinfections are also important to characterize because they can result in transmission and significant clinical disease, especially in older and vulnerable populations.

Despite the public health importance, few studies have reported rigorous estimates of reinfection risk. However, those that exist have confirmed that reinfections are relatively rare. General population estimates from Qatar, Denmark, Mexico, and the United States all found reinfection risk < 1% [7–11]. One major challenge to estimating reinfection risk is researchers rely on self-selection into testing for both first and second infections. Asymptomatic infections, accounting for at least one-third of all SARS-CoV-2 infections [12], and mildly symptomatic cases are under-represented. Data from settings that conduct regular asymptomatic testing like universities [13] and healthcare settings [14] are critical to better understand reinfection risks.

In the current study we estimated the incidence of SARS-CoV-2 reinfection among students, faculty, and staff at Indiana University (IU) across the 2020–2021 academic year. We also contextualized the observed reinfections with demographic and infection data.

## Methods

**Fig. 1:**
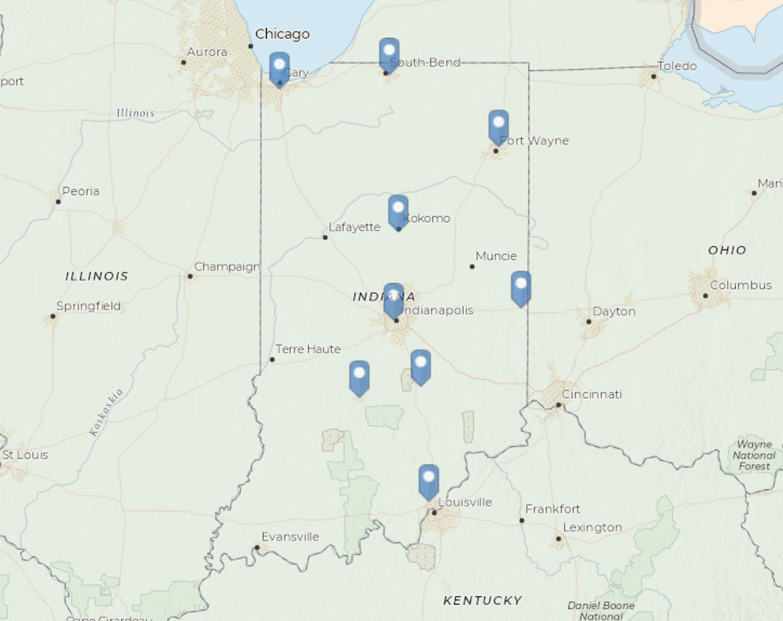
Locations of the nine Indiana University campuses with student, faculty, and staff contributing SARS-CoV-2 infection data

This study was conducted using data from IU, a large, public university with nine campuses throughout Indiana. In total, IU serves over 71,000 undergraduate and 19,000 graduate students, with over 21,000 affiliated faculty and staff. The largest enrollments are at the two core campuses of IU-Bloomington (over 45,000 students) and IUPUI (over 25,000 students). IU-Bloomington is the flagship campus located in south-central Indiana, while IUPUI is an urban campus in downtown Indianapolis. The seven additional campuses are spread across the state of Indiana (see Fig. 1) and represent smaller enrollments (generally 1000–5000). Overall, 70% IU students are Indiana residents, and 27% are from diverse racial/ethnic backgrounds (multiracial, African American, American Indian, Asian American, Hispanic, or Pacific Islander).

Upon reopening in Fall 2020, IU put in place key COVID-19 prevention measures, including a comprehensive SARS-CoV-2 testing program, mandatory masking, 6-foot distancing requirements, cleaning and disinfecting protocols, and consolidation of the academic calendar to limit student travel. The comprehensive testing program began in August 2020 and included mandatory arrival testing for students, on-demand symptomatic testing for all IU affiliates, and large-scale asymptomatic surveillance testing for all IU affiliates.

The surveillance testing involved administering saliva-based RT-PCR tests to determine weekly infection status of a large sample of affiliates (14,000 to 20,000 tests per week). During the 2020–2021 academic year, surveillance testing accounted for the vast majority of all tests recorded in the IU database (83%, see Additional file 1: Table S1). For the surveillance testing, IU followed the University of Illinois covidSHIELD protocol, which reports a performance of 96% sensitivity and 99% specificity for the detection of nucleic acid from SARS-CoV-2 in saliva samples [15]. The decision to choose saliva-based testing over nasopharyngeal swab-based testing was made because, with nasopharyngeal sampling, the throughput was slower, it required 1:1 trained personnel for collection, and it was supply-dependent at a time when swab shortages were common. Saliva-based testing allowed the university to test at a larger scale with minimal supplies and personnel, and the testing mode was less invasive and generally preferred among people who needed to be retested regularly.

Stratified random sampling allowed for more tests in demographic groups at higher risk for infection (i.e. on-campus undergraduate students), while maintaining random selection within groups. The full population of IU affiliates was categorized by campus and by type of affiliation (student, faculty, staff). Students were further subdivided into undergraduate and graduate students, into those with on-campus vs. off-campus residences, and into those with affiliations (eg. Greek organizations, athletics) that may have placed them at heightened risk for transmission. Each week, each group was assigned a probability of being selected for testing, based on their risk profile and the total testing capacity available. Generally, undergraduate students, residential students, and students affiliated with Greek organizations were assigned a higher probability of being selected for testing than other groups. However, with few exceptions (e.g. fully remote workers or students with no campus interaction), all IU affiliates were in the sampling frame for random surveillance testing each week.

Results for all SARS-CoV-2 tests, either university administered or self-reported by individuals, were recorded in an administrative database. Additional file 1: Table S1 reports the total number of test results in the database, by test type. For this study, we created an analytic cohort of all IU students, faculty, and staff with at least one SARS-CoV-2 test during the 2020–2021 academic year (August 1, 2020 to May 9, 2021). All people contributed person-time to the cohort for first infections starting August 1, 2020. For reinfection outcomes, people contributed person-time starting 12 weeks after first infection. People were censored at the time of positive test or May 9, 2021. Because our outcome of interest was SARS-CoV-2 infections, we also censored anyone who received a COVID-19 vaccine under observation, 2 weeks after their final vaccination. Of our total population, n = 22,465 (26.78%) were fully vaccinated before the study end date of May 9, 2021 and censored. This number was even lower for the cohort contributing person-time to the reinfection analysis (n = 1906; 15.53%). The median date of full vaccination was April 10, 2021 (IQR 66 days), less than 1 month before the end of our observation period. The dataset we used was deidentified prior to analysis. The IU Human Subjects Office deemed the study ‘Not Human Subjects Research’ (#11844).

Our primary outcome of interest was incident SARS-CoV-2 reinfection. We defined this outcome as any positive SARS-CoV-2 test at least 12 weeks after a first positive test. As a point of comparison, we also calculated the rates of first infection, defined as the first positive SARS-CoV-2 test under observation.

We contextualized the reinfection data with information on key covariates: age (< 30, 30–40, and > 40 years), sex (male/female), Greek organization membership (yes/no), and type of IU affiliation (student/faculty/staff). We also categorized by testing type (asymptomatic/symptomatic) to make some inference about the symptomology of first infections relative to reinfections. Finally, we calculated the median date of infection for those with single infections and those with reinfections (separately for 1st and 2nd infections).

We calculated incidence rates and 95% confidence intervals for reinfections and first infections directly. We produced a Kaplan–Meier survival curve for the reinfection cohort to visually represent the timing of reinfections. The Kaplan–Meier survival curve graphs the probability of remaining free from reinfections over the course of follow-up. We compared the profiles of people with single infections vs. reinfections using Chi-square tests for the categorical socio-demographic variables and Mann–Whitney U tests for the continuous date and time interval variables.

## Results

**Fig. 2:**
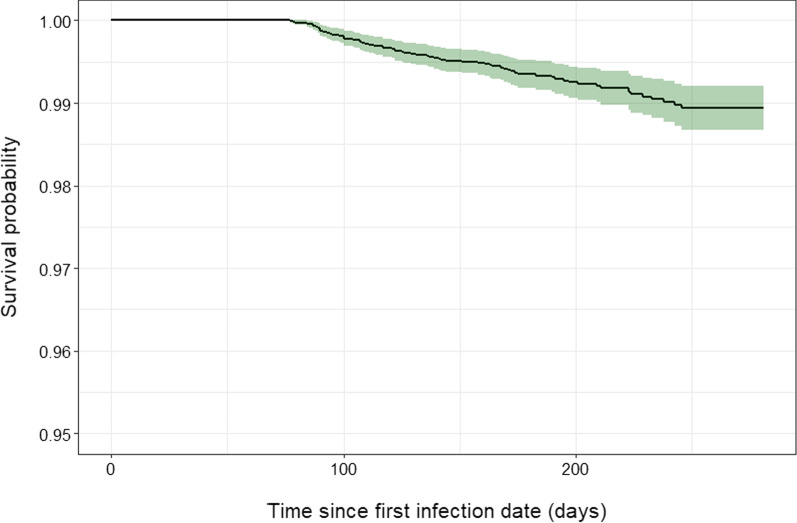
Kaplan–Meier survival curve for SARS-CoV-2 reinfection in n = 12,272 Indiana University affiliates with first infections between August 1, 2020 to May 9, 2021

Overall, 83,878 IU affiliates contributed at least one RT-PCR test in the 2020–2021 academic year (Table 1). This population was majority under age 30 (74%), female (55%), and/or student (79%). Just under one-tenth of the population was a member of a Greek organization (8.3%). Overall, 12,272 (14.6%) of affiliates tested positive for SARS-CoV-2 during that time. Of those, 74 (0.6%) went on to experience a SARS-CoV-2 reinfection. Kaplan–Meier survival estimates underscore the low risk of reinfection with 98.9% of the uncensored population remaining free from reinfection at the end of the 9-month study period (Fig. 2). Visual inspection of the curve suggests that after the wash-out period of 12 weeks, the reinfections that were observed occurred at a fairly steady rate.

**Table 1:** Characteristics of the Indiana University community with testing data from August 2020–May 2021, stratified by single infection and reinfection status

Demographic characteristics	Total population (N = 83,878)^a^	Single infection without re-infection (N = 12,198)	Reinfection (N = 74)		p value (reinfection vs single)^b^
			First infection	Second infection	
Age (years), N (%)					0.1
< 30	62,205 (74.2)	10,731 (88.0)	70 (94.6)		
30–40	9299 (11.1)	625 (5.1)	3 (4.0)		
> 40	12,374 (14.7)	842 (6.9)	1 (1.4)		
Sex, N (%)					0.9
Male	37,607 (44.8)	5637 (46.2)	35 (47.3)		
Female	46,269 (55.2)	6561 (53.8)	39 (52.7)		
Unknown	2 (0.0)	0 (0)	0 (0)		
Greek organization membership, N (%)					0.001
No	76,905 (91.7)	9072 (74.4)	42 (56.8)		
Yes	6973 (8.3)	3126 (25.6)	32 (43.2)		
Greek organization type (among Greek members), N (%)					0.7
Fraternity	3147 (45.1)	1514 (48.4)	14 (43.7)		
Sorority	3826 (54.9)	1612 (51.6)	18 (56.3)		
IU affiliation status, N (%)					0.1
Student	65,831 (78.5)	11,013 (90.3)	71 (96.0)		
Staff	10,685 (12.7)	875 (7.2)	1 (1.4)		
Faculty	7362 (8.8)	310 (2.5)	2 (2.7)		
Positive test type, N (%)					0.1
Asymptomatic^c^	–	5584 (45.8)	41 (55.4)	41 (55.4)	
Symptomatic^d^	–	6614 (54.2)	33 (44.6)	33 (44.6)	
Median date of infection (IQR)	–	11/11/2020 (9/10/2020, 1/2/2021)	9/8/2020 (9/1/2020, 11/2/2020)	2/25/2021 (12/30/2020, 3/29/2021)	< 0.0001
Median time interval (days) between 1st and 2nd infection (IQR)	–	–	–	123.5 (98.3, 171.8)	–

^a^Total population includes all members of IU affiliated faculty, staff, and students with at least one SARS-CoV-2 test during the study period of August 1, 2020 to May 9, 2021

^b^p-values for categorical variables are from Chi-square tests. p-values for continuous variables are from Mann–Whitney U tests

^c^Asymptomatic tests include the following test categories: surveillance, voluntary, and arrival tests. The total number of tests in these categories were 4363, 770, 451, respectively, for single infections; 33, 3, 5, respectively, for the first diagnostic test of reinfected population; and 31, 10, 0, respectively, for the second test of reinfected population

^d^Symptomatic tests include the following test categories: Self-report, symptomatic, and other tests. The total number of tests in these categories were 4795, 1818, 1, respectively, for single infections; 26, 7, 0, respectively, for the first diagnostic test of reinfected population; and 30, 3, 0, respectively, for the second test of reinfected population

The incidence rate for reinfections [0.4 per 10,000 person-days (95% CI 0.3, 0.5)] was low relative to the incidence rate for first infections [6.0 per 10,000 person-days (95% CI 5.9, 6.1)]. The median date of infection for single infections (November 11, 2020) was significantly later than the median date of first infection for those who later experienced a reinfection (September 8, 2020). The median time interval between 1st and 2nd infections was 124 days (IQR: 98–172).

Those who experienced reinfections differed from those with single infections in some notable ways. People with reinfections were significantly more likely to be members of Greek organizations. Greek members made up 8% of the total study population, 26% of the single infections, and 43% of the total reinfections (Chi-square p-value for single vs reinfections: 0.001). There were no statistically significant differences by sex, age, or type of IU affiliation. The first and second infections for people with reinfections were identified more than half of the time via an asymptomatic testing mechanism (55%), higher than observed for people with single infections (46%), though not statistically significantly different (Chi-square p-value: 0.1).

## Discussion

We found that reinfections were rare but not absent in a large population of IU students, faculty, and staff in the 2020–2021 academic year. Reinfection risk was higher in Greek-affiliated students. Because we restricted to the 2020–2021 academic year during which vaccine availability was limited, and because we censored individuals at vaccination, our results reflect reinfections in the context of natural immunity.

Our findings closely align with the few existing estimates of reinfection risk, despite the varied settings and designs used. In datasets with limited asymptomatic testing in Qatar, Denmark, Mexico, and the US, the reinfection risk ranged from 0 to 0.65%, with variable follow-up time ranging from 5 to 10 months [7–11, 14]. Our risk estimate of 0.6% is in line with the upper level of these estimates. It is lower than the single other study of reinfections in a university setting with regular asymptomatic testing. That study, from Clemson University over the same study period, found a reinfection rate of 2.2% [13]. The Kaplan–Meier survival estimates (98.9% at IU vs. 97.2% at Clemson) and median durations between infections (124 at IU vs. 129 days at Clemson) between our two study populations are notably similar.

The observed higher risk of reinfection among Greek-affiliated students deserves special attention. This finding may reflect a true increased risk underlying a population that has been characterized by high levels of alcohol consumption, large social events, and congregate living. Early evidence suggests high-risk alcohol consumption is associated with SARS-CoV-2 infections [16], with plausible mechanisms through the loss of inhibitory control [17] and damage to the immune system [18]. However, our findings are also likely influenced by ascertainment bias, with Greek-affiliated students being required to test more frequently than some other groups, leading to an increased likelihood of identifying asymptomatic reinfections. The outbreak dynamics on IU campuses may have also led to selection bias in length of follow-up time for this group. There were large SARS-CoV-2 outbreaks in Greek-affiliated students at the beginning of the Fall 2020 semester, aligned with our earliest possible follow-up time. Thus, Greek-affiliated students tended to have more person-time at risk for the reinfection outcome, relative to individuals who experienced their first infection later in the follow-up period.

There are several aspects of our data that warrant caution in interpreting our findings. Importantly, given the rarity of the reinfection outcomes, many of our estimates were measured imprecisely. This is true even given the very large total population (n > 80,000) that underpins these estimates. Future studies should consider pooling data across universities with similar testing schemes to maximize statistical power. Also, it is possible that some reinfections were misclassified. To address this concern, we included a 12-week wash-out period between positive tests to determine reinfection status. However, we did not require laboratory-confirmed negative RT-PCR results between infections, or otherwise determine distinct infections with phylogenetic analyses. Thus, it is possible for viral remnants to account for some of the reinfections, though unlikely given the length of washout period we applied. In the other direction, our testing system did not test every IU affiliate at regular, frequent intervals, nor did we collect complete information on positive tests prior to August 2020. Thus, some true reinfections could have been misclassified as single infections. However, this concern is somewhat alleviated by the fact that the time period under observation was fairly early in the pandemic, and by data from a serological study among IU-Bloomington undergraduates revealing low seropositivity (4.6%) at the beginning of the study period [19].

## Conclusions

In sum, we found evidence for low levels of SARS-CoV-2 reinfection in a large multi-campus university population during a time-period prior to widespread COVID-19 vaccination. These findings suggest that prior infections should not exempt people from surveillance and mitigation efforts, and that people with prior infections should not rely on natural immunity to protect against subsequent infection. Instead, given the strong evidence that full vaccination does protect against reinfection [20], people with prior infections should be encouraged to become fully vaccinated.

## Supplementary Information

**Additional file 1: Table S1.** Distribution of Indiana University SARS-CoV-2 tests by type, August 2020–May 2021.

## Data Availability

The dataset used in this study is not publicly available due to the sensitive nature of the infection data. The corresponding author will help facilitate data access upon reasonable request.
