# Supplementary material for: SARS-CoV-2 reinfections in a US university setting, Fall 2020 to Spring 2021
Source: BMC Infect Dis. 2022 Jul 4;22:592. doi: 10.1186/s12879-022-07578-x (PMC9252534; doi:10.1186/s12879-022-07578-x)
Supplement: Supplementary file 1 — Additional file 1: Table S1. Distribution of Indiana University SARS-CoV-2 tests by type, August 2020–May 2021. [file 12879_2022_7578_MOESM1_ESM.docx]

**SUPPLEMENTARY MATERIAL**

**Table S1.** Distribution of Indiana University SARS-CoV-2 tests by type, August 2020-May 2021

| **Test type** | **Number of tests (%)** |
| --- | --- |
| Antigen | 12,661 (1.7) |
| Arrival | 38,574 (5.2) |
| Surveillance | 621,088 (83.2) |
| Other | 711 (0.1) |
| Pre-Arrival | 11,656 (1.6) |
| Self-Report | 5,143 (0.7) |
| Symptomatic | 8,418 (1.1) |
| Voluntary | 47,888 (6.4) |
| **TOTAL** | **746,139** |
